# Technical Notes: Robustness of three‐dimensional treatment and imaging isocenter testing using a new gel dosimeter and kilovoltage CBCT

**DOI:** 10.1002/acm2.14439

**Published:** 2024-06-20

**Authors:** Riki Oshika, Hidenobu Tachibana, Kazuya Seki, Rie Tachibana, Shunsuke Moriya, Takeji Sakae

**Affiliations:** ^1^ Degree Programs in Comprehensive Human Sciences Graduate School of Comprehensive Human Sciences University of Tsukuba Ibaraki Japan; ^2^ Radiation Safety and Quality Assurance division National Cancer Center Hospital East Chiba Japan; ^3^ Triangle Products Co. Ltd. Chiba Japan; ^4^ Faculty of Medicine University of Tsukuba Ibaraki Japan

**Keywords:** dGEL™, gel dosimetry, robustness, 3D starshot, X‐ray CT‐based gel dosimeter

## Abstract

**Background:**

Coincidence of the treatment and imaging isocenter coordinates is required to safely perform small‐margin treatments, such as stereotactic radiosurgery of multiple brain metastases. A comprehensive and direct methodology for verifying concordance of kilovoltage cone‐beam computed tomography (kV‐CBCT) and treatment coordinates using an x‐ray CT‐based polymer gel dosimeter (dGEL) and onboard kV‐CBCT was previously reported. Using this methodology, we tested the ability of a new commercially available x‐ray CT‐based polymer dGEL with a rapid response to provide efficient quality assurance (QA).

**Purpose:**

The aim of this study was to evaluate the robustness of the three‐dimensional geometric QA methodology using dGEL.

**Methods:**

The dGEL were commercially manufactured. The prescribed dose for each field was determined by visually identifying the 5, 10, and 20 Gy isodose lines. A linear accelerator was used to irradiate the gels with seven non‐coplanar beams. An in‐house analysis program was used to identify the beam axes and treatment isocenter in kV‐CBCT coordinates by processing the pre‐ and post‐irradiation CBCT images. The impact of the radiation dose on the test reproducibility was examined, and the detectability of an intentional geometric error was assessed.

**Results:**

The treatment isocenter was within 0.4 mm of the imaging isocenter for all radiation doses. The residual error of the test with the intentional error was within 0.2 mm. The analysis and image quality variations for a single dGEL introduced displacement errors less than 0.3 mm.

**Conclusions:**

The test assessed the coincidence of treatment and kV‐CBCT isocenter coordinates and detected errors with high robustness. Even for a 10 Gy dose, the test yielded results comparable with those obtained using higher radiation doses owing to the rapid response of the dGEL dosimeter.

## 1 INTRODUCTION

Onboard kilovoltage cone‐beam computed tomography (kV‐CBCT) plays an increasing role in creating an accurate patient setup for stereotactic radiosurgery (SRS) and stereotactic radiotherapy (SRT).^1^ Coincidence of the treatment and imaging isocenter coordinates is required to safely perform small‐margin treatments such as SRS and SRT.^2^ The American Association of Physicists in Medicine task group reports 142 and 198 recommend that the treatment and imaging isocenter coordinates deviate by ≤ 1 mm for an SRS/SRT linear accelerator.^3^, ^4^ The spatial accuracy of the treatment and imaging isocenter coordinates is evaluated using several quality assurance (QA) measures, such as the Winston–Lutz test^5^ and star‐shot test.^6^ These tests do not directly evaluate the coincidence of the treatment and kV‐CBCT imaging isocenters but rather the coincidence of mechanical surrogates such as a metallic ball and an external laser, respectively.

Pant et al. proposed a three‐dimensional (3D) isocenter verification test using an *N*‐isopropylacrylamide (NIPAM) polymer gel dosimeter for evaluation of treatment and kV‐CBCT isocenters.^7^ [Correction added on July 26 2024, after first online publication: (dGEL) in parentheses was removed from the sentence.] This approach enables a comprehensive 3D evaluation of radiation accuracy (isocentricity and coincidence) relative to the imaging isocenter. The trajectories of radiation beams through the dosimeter are visualized using CBCT, with the imaging isocenter being directly recorded. Thus, the isocentricity and the coincidence between the treatment and imaging isocenters were directly assessed. Additionally, because QA phantoms with laser alignment are not necessary, human errors in their setup do not affect the accuracy of the test.

There are some problems with the above methodology. First, it requires a relatively large radiation dose from each beam to visualize the beam axis on the CBCT image. Pant et al. used approximately 16 Gy per beam for the NIPAM dosimeter,^7^ and Kozicki and Maras used 10 000 monitor units (MU) for the PABIG^nx^ dosimeter.^8^ Second, a long waiting time between irradiation and imaging is required. For example, Johnston et al.^9^ reported that one must wait 24 h after irradiation before the NIPAM dosimeter can be used to visualize the trajectories on a CBCT image. However, it was shown elsewhere^10^ for the same NIPAM dosimeter, and other dosimeters, that the irradiation, scanning, and data processing takes about 45 min. Moreover, such visualization may need an incremented CT number of at least 5 HU. No change in the CT number of the NIPAM dosimeter was measured immediately after irradiation according to one study.^9^ However, in another work, NIPAM and other dosimeters were measured just after irradiation.^10^ For the PABIG^nx^ dosimeter,^8^ the CT number was 4 HU higher immediately after 10 000 MU irradiation and 10 HU higher 20 h after irradiation. In the study, the CT scan of the dosimeter was performed just after irradiation. To obtain a sufficient increase in CT number (ΔHU) for the test, a high radiation dose is required (The irradiation time can be reduced by using high MU rates). Moreover, only some dosimeters must be stored in a refrigerator until imaging and irradiation.^7^, ^9^ Especially, the NIPAM dosimeter was stored in the refrigerator until imaging and irradiation (4 days on average) in a study.^7^ But there is limited access to refrigerators dedicated to this purpose in clinical radiation departments.

**FIGURE 1:**
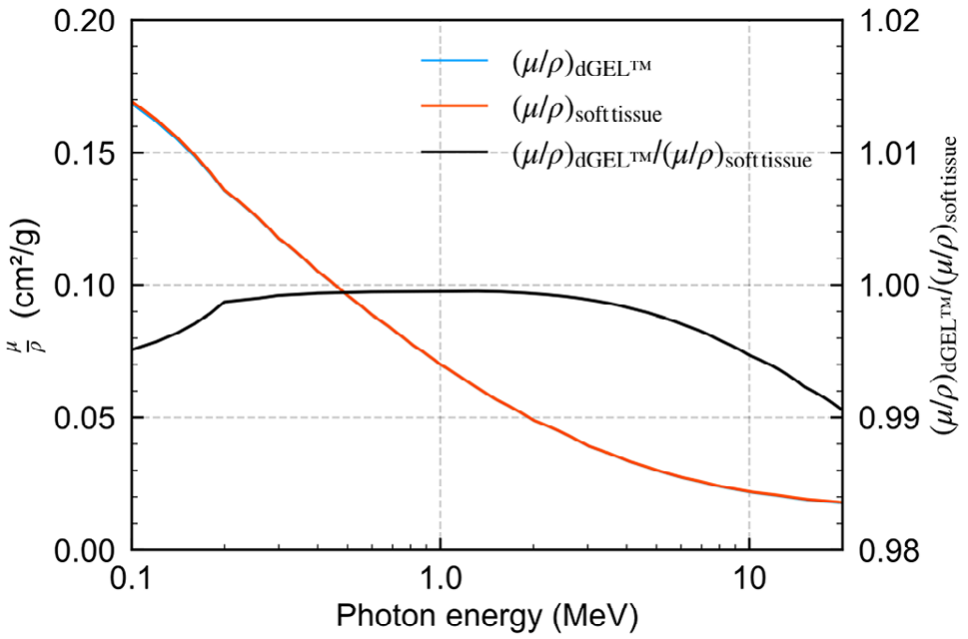
Mass attenuation coefficients for dGEL and soft tissue (ICRU Report 44^11^) and their ratio. μ/ρ, mass attenuation coefficient. dGEL, gel dosimeter.

**FIGURE 2:**
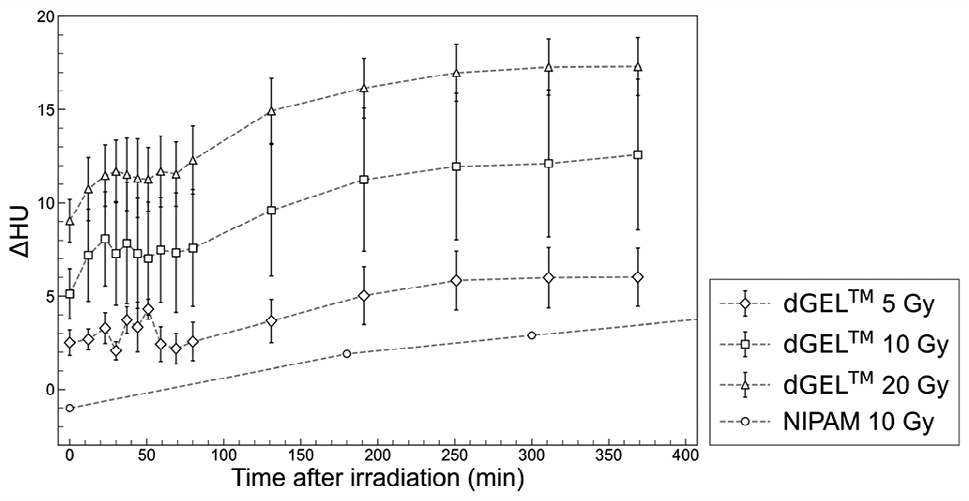
The increase of CT number (ΔHU) of dGEL measured at post‐irradiation times of 0–360 min in a study by Tachibana et al.^12^ The results are compared with those obtained using an NIPAM‐based gel in a study by Johnston et al.^9^ The error bars indicate the standard deviation. dGEL, gel dosimeter; NIPAM, *N*‐isopropylacrylamide.

Recently, a new x‐ray CT‐based polymer gel dosimeter called dGEL was developed. [Correction added on July 26 2024, after first online publication: the word ‘polymer’ was updated to ‘polymer gel dosimeter’ in the sentence.] Commercially manufactured by Triangle Products Co., Ltd. (Kashiwa, Japan), its ΔHU increases rapidly after irradiation, and it remains in the solid state even at room temperature (approximately 24°C). The dGEL has near‐tissue equivalence and is based on a polymer gel composition. In Table 1, the elemental composition and mass density of dGEL are compared to those of soft tissue (ICRU Report 44^11^). Figure 1 shows a comparison of mass attenuation coefficient between dGEL and soft tissue (ICRU Report 44^11^). Tachibana et al.^12^ showed that the response of the dGEL dosimeter was more rapid than that of a NIPAM gel, as shown in Figure 2. The rapid response of the new dGEL improves upon the results of Pant et al. to provide a clinically feasible QA method. Although Pant et al. used a relatively high dose of approximately 16 Gy for a single beam, the minimum dose required for analysis using the dGEL dosimeter is not yet known.

**TABLE 1:** Elemental composition, mass density, electronic density, and effective atomic number of the dGEL dosimeter and soft tissue (ICRU Report 44^11^).

	Elemental composition (% by weight)											
	H	C	N	O	Na	P	S	Cl	K	ρ (g/cm^−3^)	$\rho_{e}/N_{A}$	$Z_{\mathrm{eff}}$
dGEL	10.2	17.5	3.9	68.3	–	0.0131	0.0068	–	–	1.02	0.563	7.23
Soft tissue (ICRU 44)	10.2	14.3	3.4	70.8	0.2	0.3	0.3	0.2	0.3	1.06	0.583	7.64

*Note*: ρ, mass density; $\rho_{e}$, electronic density; $N_{A}$, Avogadro constant; $Z_{\mathrm{eff}}$, effective atomic number.

Abbreviation: dGEL, gel dosimeter.

The methodology proposed by Pant et al.^7^ provides a 3D geometric irradiation accuracy assessment without human errors, and an error‐free result is expected to enable more precise radiotherapy by enabling more accurate margin determination. However, medical physicists may have concerns about implementing the new methodology for QA because of unexpected instabilities in the analysis using the dGEL dosimeter. In particular, the method in the software to find the trajectories of radiation beams through the dosimeter may find a local rather than global minimum because it uses optimization calculations based on the contrast‐to‐noise ratio (CNR).

In this study, we assessed the robustness of the methodology for verifying the coincidence of the treatment and kV‐CBCT isocenter coordinates using the new dGEL dosimeter and evaluated the detectability of geometric errors and reproducibility of the test.

## 2 METHODS

### 2.1 Dosimeter preparation

The new dosimeter was manufactured by Triangle Products Co., Ltd. (Kashiwa, Japan). Each gel solution was poured into a 500 mL cylindrical jar made of poly(vinyl chloride) with a diameter of 8.0 cm and height of 14.5 cm. To minimize oxygen effects, mineral oil was added to the top of each jar to displace the air above the gel, and the top of the jar was covered with parafilm prior to capping. The gel‐containing jar was placed in an incubator set to a temperature of 24°C, and 24 h after manufacture the jar was sent to our institution in an expanded polystyrene container.

### 2.2 Treatment planning

**FIGURE 3:**
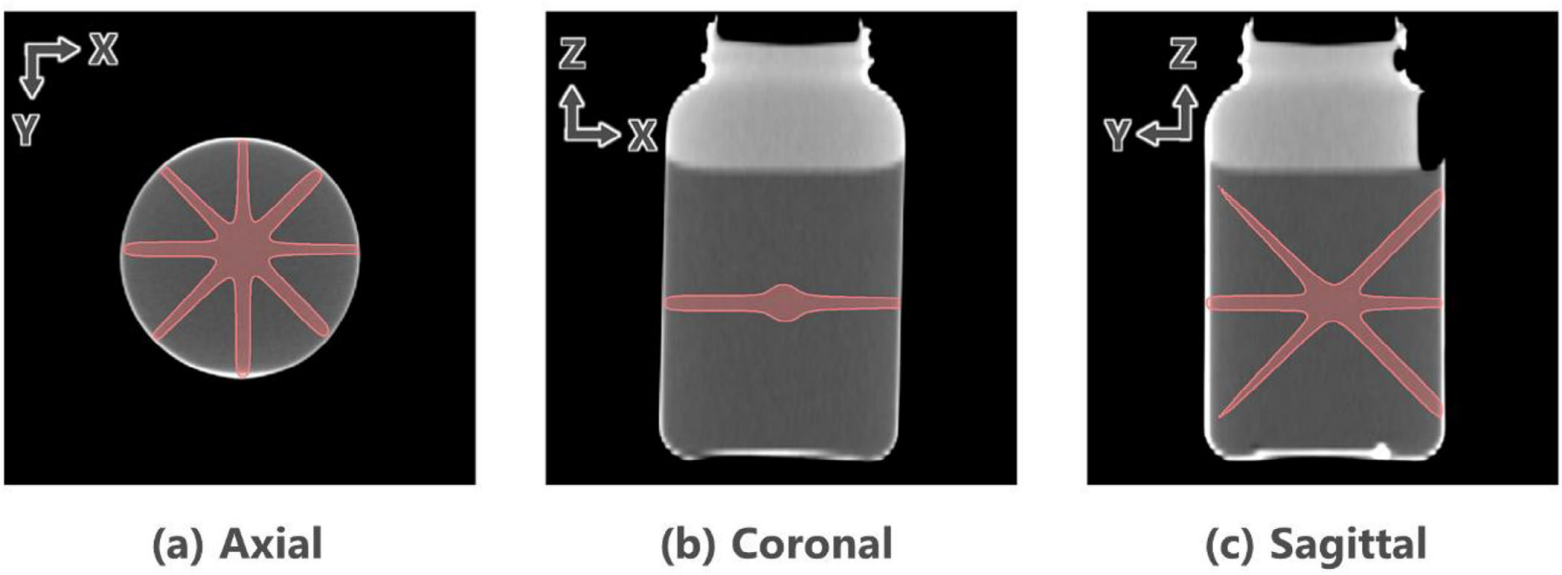
Planned dose distribution for the isocenter verification test using the dGEL. The color wash represents a radiation dose of 20 Gy. dGEL, gel dosimeter.

All treatment plans were created using version 15.6 of the AcurosXB dose calculation algorithm implemented in the Eclipse treatment planning system (Varian Medical Systems, Palo Alto, CA, USA) and a dose calculation grid of 2 mm. All treatment plans used a common CT dataset acquired using an Acquilion ONE system (Canon Medical Systems, Otawara, Japan) with the following image acquisition and reconstruction parameters: tube voltage, 120 kVp; tube current, 300 mA; resolution, 0.78 mm/pixel; slice thickness, 2.0 mm. Seven non‐coplanar radiation beams with a field size of 5 × 5 mm^2^ set by collimator jaws were delivered at unique gantry and couch angles (Table 2) and passed through the isocenter. [Correction added on July 26 2024, after first online publication: the acronym for couch angles, (CA), was deleted from the sentence.] Three different plans were created, with each beam delivering doses of either 5 Gy (plan 1), 10 Gy (plan 2), or 20 Gy (plan 3). Figure 3 shows the isodose lines of the 20 Gy radiation dose in three different slices. MUs were adjusted such that the isodose line corresponded to the above three doses.

**TABLE 2:** Gantry angle and couch angle used for the verification test. [Correction added on July 26 2024, after first online publication: the word ‘GA and CA’ is updated to ‘Gantry angle’ and ‘couch angle’.]

Field no.	Gantry angle (°)	Couch angle (°)
1	45	0
2	135	0
3	180	0
4	270	0
5	45	45
6	45	90
7	45	270

### 2.3 Irradiation and CBCT image acquisition

**FIGURE 4:**
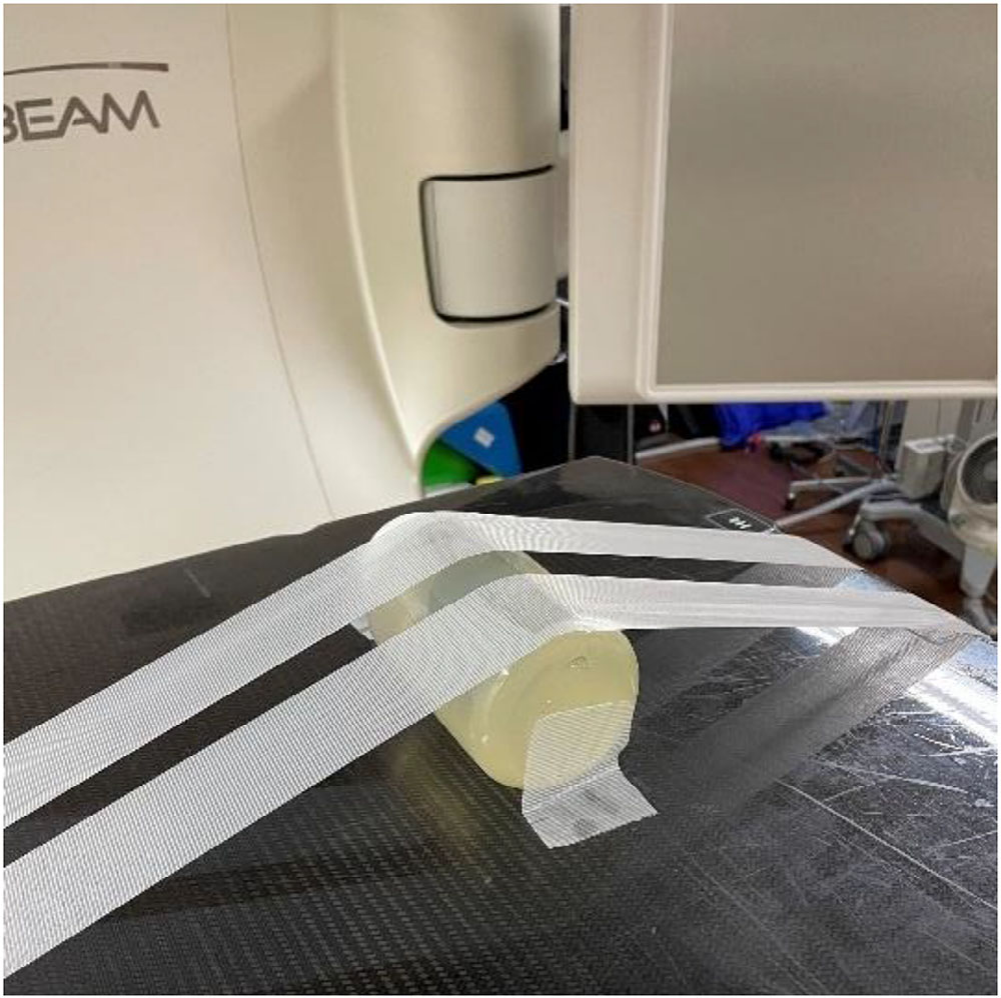
Positioning of the dGEL. dGEL, gel dosimeter.

All polymer gels were irradiated using a TrueBeam linear accelerator (Varian Medical Systems). The machine dose rate was 2400 cGy min^−1^, and the energy of each flattening‐filter‐free photon beam was 10 MV to reduce the irradiation time. Dose rate (cGy min^−1^) is defined as the absorbed dose (cGy) delivered per minute to a depth of maximum dose within a 10 × 10 cm^2^ treatment field. The dosimeter was placed with the center of its sensitive volume located approximately at the mechanical isocenter indicated by the room laser without CBCT‐based localization. The dGEL was placed on the couch and secured in place using adhesive tape to prevent movement (Figure 4). Before and immediately after irradiation, CBCT images of the gel were acquired using an onboard kV‐CBCT system attached to the linear accelerator. The pre‐ and post‐irradiation CBCT imaging was performed once for each test. The image acquisition and reconstruction parameters are shown in Table 3.

**TABLE 3:** CBCT image acquisition and reconstruction parameters.

Acquisition parameters		Reconstruction parameters	
x‐Ray voltage	80 kV	Base method	iCBCT
x‐Ray current	45 mA	Filter	Standard
x‐Ray pulse length	20 ms	Noise suppression	Medium
Frame rate	15 fps	Resolution	0.511 mm/pixel
Gantry speed	1°/s	Slice thickness	1.0 mm
Fan type	Full	Detruncation volume	Adaptive
Number of projections	5400		
Projection increment	0.4°		
Acquisition time	360 s		

Abbreviation: CBCT, cone‐beam computed tomography.

### 2.4 Analysis

#### 2.4.1 Overview

The workflow of the verification test followed the methodology of Pant et al.^7^ and was implemented using an in‐house program written in Python 3.9. The pre‐ and post‐irradiation CBCT images and DICOM‐RT plan were imported into the program to (1) define the region of interest (ROI) for analyzing each treatment beam using the dose distributions on the image and the geometry of the field; (2) identify each beam axis from the dose distributions by maximizing the CNR; and (3) determine the location of the treatment isocenter in the CBCT coordinate system. The steps of the analysis are described in detail in the following sections.

#### 2.4.2 Beam axis detection

The beam axis detection from CBCT image sets was performed in the CBCT coordinate system followed the methodology of Pant et al.^7^ The pre‐ and post‐irradiation CBCT images were used unfiltered. The ROI for the analysis of field i (analysis ROI, $V_{\mathrm{analysis},i}$) was defined as

(1) $$\begin{array}{c} \begin{array}{c} V_{\mathrm{analysis},i}=\left(V_{\mathrm{dosimeter}}\cap V_{\mathrm{field},i}\right)\cap {\left(\overset{n}{\underset{\begin{array}{c} j\\ j\neq i \end{array}}{\cup }}V_{\mathrm{field},j}\right)}^{C}, \end{array} \end{array}$$

where $V_{\mathrm{dosimeter}}$ is the dosimeter mask, which is obtained by image thresholding of the pre‐CBCT image, and $V_{\mathrm{field},i}$ is the volume irradiated by field i (field ROI), which is based on the gantry angle, couch angle, and field size obtained from the DICOM‐RT plan. [Correction added on July 26 2024, after first online publication: the acronyms GA and CA were replaced for gantry angle and couch angle in the sentence.] The threshold value was adjusted until the whole sensitive volume of the dGEL was visually identified. The beam central axis of field i, $l_{i}$, was identified from the difference in signal between the pre‐ and post‐irradiation CBCT images and mainly used to remove cupping artifacts on the CBCT image. $l_{i}$ was derived by optimizing the equation

(2) $$\begin{array}{c} \underset{l}{\arg\;\max}\mathrm{CNR}\left(l_{i}\right)=\underset{l}{\arg\;\max}\frac{C}{Z}, \end{array}$$

where C and Z are the contrast and noise, respectively:

(3) $$\begin{array}{c} C=\frac{\sum_{k\in V_{\mathrm{analysis}}}w_{c_{k}}\left(S_{k}-\bar{b}\right)}{\sum_{k\in V_{\mathrm{analysis}}}w_{c_{k}}} \end{array}$$

(4) $$\begin{array}{c} Z=\sqrt{\frac{\sum_{k\in V_{\mathrm{analysis}}}w_{b_{k}}{\left(S_{k}-\bar{b}\right)}^{2}}{\left(n-1\right)\sum_{k\in V_{\mathrm{analysis}}}\frac{w_{b_{k}}}{n}}}. \end{array}$$

Here $S_{k}$ is the signal intensity in voxel k of the analysis ROI, $w_{c_{k}}$ and $w_{b_{k}}$ are corresponding weights for that voxel, and $\bar{b}$ is the background signal:

(5) $$\begin{array}{c} \bar{b}=\frac{\sum_{k\in V_{\mathrm{analysis}}}w_{b_{k}}S_{k}}{\sum_{k\in V_{\mathrm{analysis}}}w_{b_{k}}}. \end{array}$$

The weights are defined by

(6) $$\begin{array}{c} w_{c_{k}}=1-f\left(d_{k}-\frac{r_{\mathrm{field}}}{2},\sigma \right) \end{array}$$

(7) $$\begin{array}{c} w_{b_{k}}=f\left(d_{k}-\frac{r_{\mathrm{field}}}{2}+m,\sigma \right), \end{array}$$

where $d_{k}$ is the distance of voxel k from l, $r_{\mathrm{field}}$ is the planned field size, f(x,σ) is the normalized cumulative distribution function for the mean x and standard deviation σ, and m is an optional margin. In this study, we used m = 3.0 mm and σ = 1.0 for all analyses, and we used the sequential least squares module in SciPy (Enthought, TX, USA) for the optimization process.

#### 2.4.3 Treatment isocenter calculation

The treatment isocenter is the center of the smallest sphere through which all beam axes pass. The treatment isocenter $p_{\mathrm{treat}}=\left(x_{\mathrm{treat}},y_{\mathrm{treat}},z_{\mathrm{treat}}\right)$ is calculated from $d_{l_{i}}\left(p_{\mathrm{treat}}\right)$, the distance between each beam axis $l_{i}$, using

(8) $$\begin{array}{c} \underset{p_{\mathrm{treat}}}{\arg\;\min}r\left(p_{\mathrm{treat}}\right)=\underset{p_{\mathrm{treat}}}{\arg\;\min}\left(\max_{i}d_{l_{i}}\left(p_{\mathrm{treat}}\right)\right). \end{array}$$

### 2.5 Assessment of the isocenter verification test

#### 2.5.1 Impact of radiation dose

To assess the impact of radiation dose, the test was performed for each of the three treatment plans.

#### 2.5.2 Detectability of an intentional error

A test was performed with an intentional geometric error of −0.5, 0.5, and −0.5 mm in the respective *X*, *Y*, and *Z* directions of the DICOM coordinate system. This error was generated by adjusting the offset between the treatment isocenter and the couch. The radiation dose for this test was 20 Gy per field.

#### 2.5.3 Reproducibility of the verification

To understand the robustness of the methodology, the analysis reproducibility, and test reproducibility were assessed. Analysis reproducibility was assessed by comparing program outputs from the optimization process during a three‐time analysis of the same CBCT dataset. To assess test reproducibility, outputs from the optimization process for three‐time scanned CBCT datasets of the same irradiated gels were compared. Here, three pre‐CBCT and three post‐CBCT scans were performed using a dosimeter irradiated with 20 Gy per field. From these, nine combinations of CBCT image pairs were analyzed using the program.

#### 2.5.4 Comparison with the conventional isocenter verification test

The agreement between treatment and imaging isocenters in the 20 Gy test was compared with that determined using conventional Winston–Lutz test,^5^ which measures the alignment of a metallic ball in kV‐CBCT. The test was performed using a cube phantom containing a central metal ball (Varian Medical Systems, Palo Alto, CA, USA). The cube phantom was aligned using the CBCT image, and the image location containing the center of the cube corresponded to the imaging isocenter. Subsequently, four planar images were obtained using a 2.5 MV x‐ray beam with gantry angles of 0°, 90°, 180°, and 270° and then analyzed using Simple QA Analysis software (Triangle Products Co., Ltd. Kashiwa, Japan) to determine the distance between the treatment isocenter and cube center. [Correction added on July 26 2024, after first online publication: the acronym GA was replaced for gantry angles in the sentence.]

## 3 RESULTS

### 3.1 Treatment and imaging isocenter verification

**FIGURE 5:**
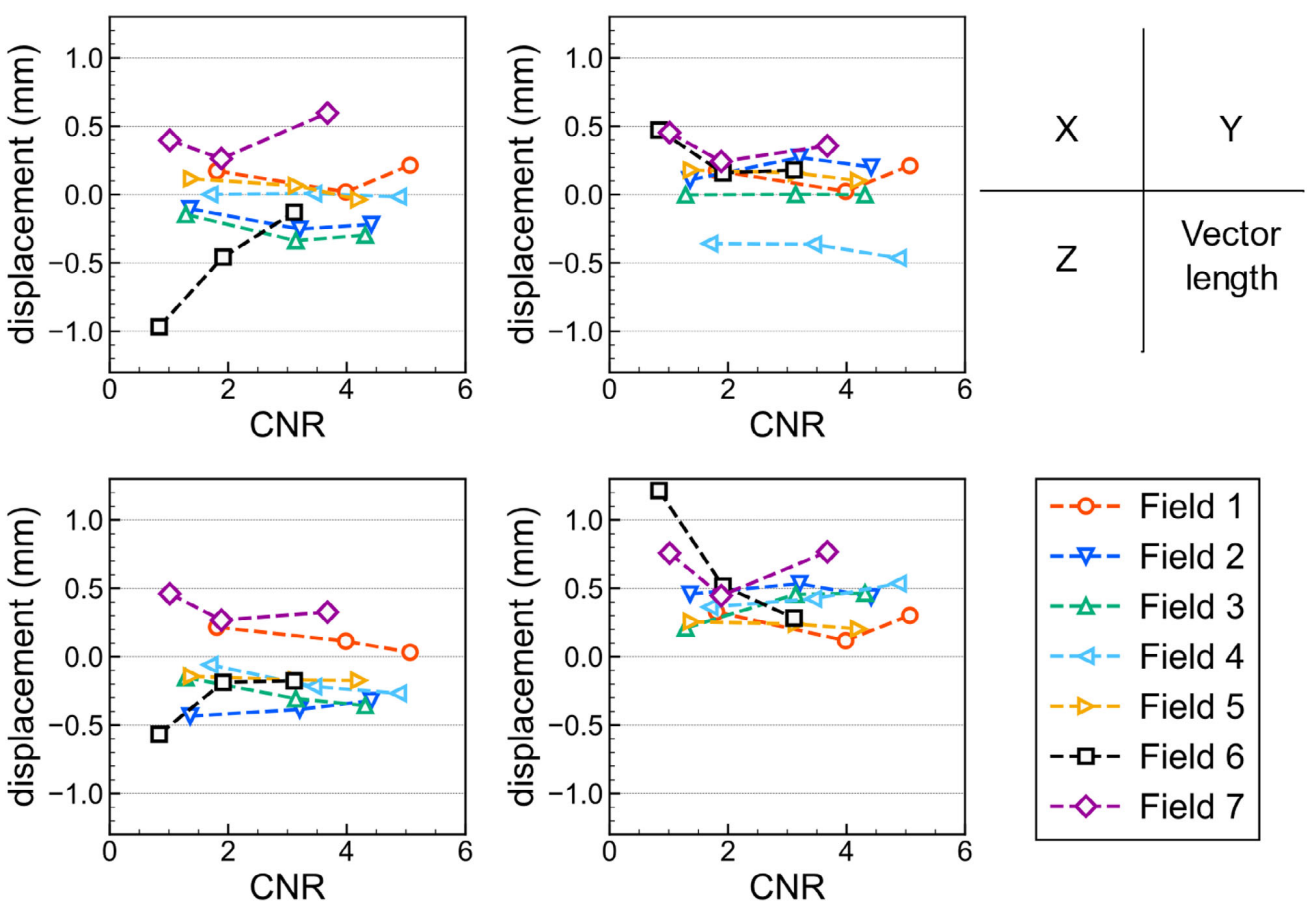
Displacements of the beam axis from the imaging isocenter as a function of the CNRs. CNRs, contrast‐to‐noise ratios. [Correction added on July 26 2024, after first online publication: the figure 5 caption has been updated.]

Table 4 shows the displacement of the treatment isocenter from the imaging isocenter and the smallest radius that intersects all beams, determined using the 3D isocenter verification test for each radiation dose. Displacements in the *X*, *Y*, and *Z* directions were measured, and the length of the displacement vector directed from the treatment isocenter to the imaging isocenter was calculated. The mean CNRs were 1.34, 2.96, and 4.24 for radiation doses of 5, 10, and 20 Gy, respectively; and the total irradiation times, which included the pre‐ and post‐irradiation CBCT image acquisition time, were 20, 25, and 36 min, respectively. The greatest displacement was measured for the prescribed dose of 5 Gy, which produced the lowest CNR. However, the displacements for all radiation doses were less than 0.4 mm. Figure 5 shows the displacement of the beam axis from the imaging isocenter as a function of the CNR. The displacement among different CNRs differed by less than 0.3 mm for all beams except No. 6. The length of the displacement vector for the prescribed dose of 5 Gy with the lowest CNR (0.83) showed the greatest deviation (0.7 mm) from the vector lengths measured for the other doses.

**TABLE 4:** Displacement of the treatment isocenter from the imaging isocenter and smallest radius that intersects all beams, determined using the 3D verification test.

	Displacement from imaging isocenter (mm)				
Dose (Gy)	*X*	*Y*	*Z*	Vector	Smallest radius that intersects all beam (mm)
5	–0.28	0.43	–0.08	0.53	0.79
10	–0.03	0.06	–0.05	0.08	0.46
20	0.23	0.09	–0.09	0.27	0.60

Abbreviation: 3D, three dimensional.

### 3.2 Detection of an intentional error

**FIGURE 6:**
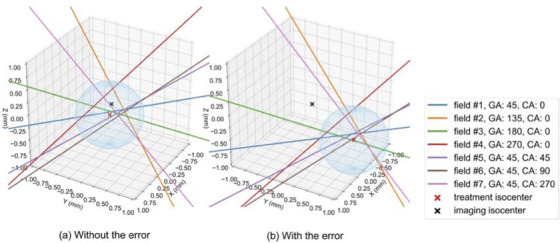
Beam axes and treatment isocenters reconstructed in 3D space with and without an intentional error. The blue sphere indicates the area of the treatment isocenter. 3D, three dimensional; GA, Gantry angle; CA, Couch angle.

Table 5 shows the displacements in the tests with and without an intentional error, and the corresponding images are shown in Figure 6. The vector length of the residual error was less than 0.2 mm.

**TABLE 5:** Results of the verification when an intentional error was introduced.

	No error applied	Intentional error applied		
	Displacement	Displacement	Couch offset	Residual error
*X* (mm)	0.23	–0.33	–0.50	0.17
*Y* (mm)	0.09	0.67	0.50	0.17
*Z* (mm)	–0.09	–0.66	–0.50	–0.16
Vector (mm)	0.27	1.00	0.87	0.13

### 3.3 Reproducibility of the verification

**FIGURE 7:**
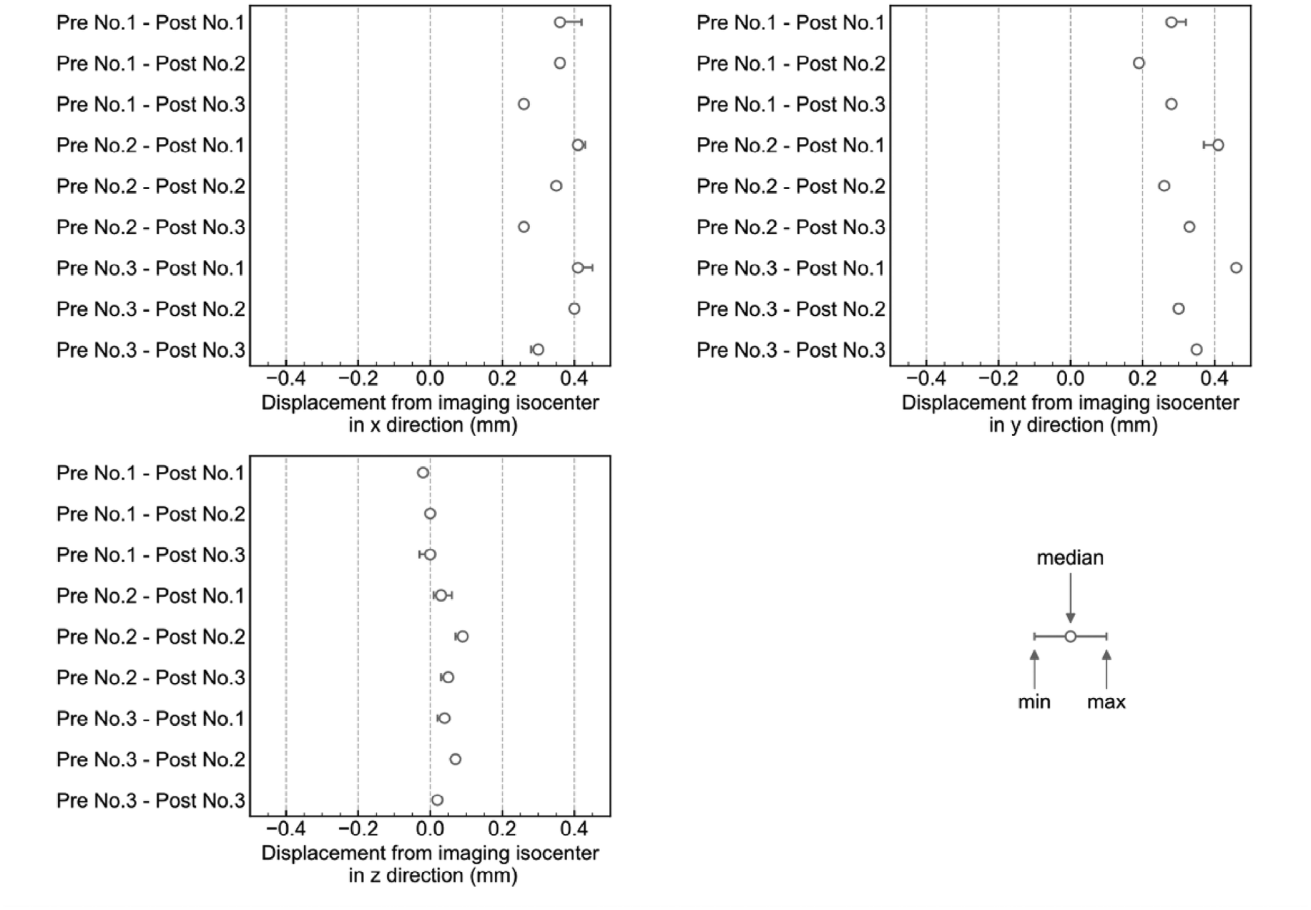
Treatment isocenter displacement from the imaging isocenter for nine pre‐ and post‐irradiation CBCT image combinations obtained from three imaging analyses of the same dosimeter. CBCT, cone‐beam computed tomography.

Figure 7 plots the displacement of the treatment isocenter from the imaging isocenter for nine combinations of pre‐ and post‐irradiation CBCT images obtained from three analyses of a single dosimeter. The range between the maximum and minimum displacements provides a measure of analysis reproducibility. Variations between the nine combinations provide a measure of test reproducibility. The analysis reproducibility was within 0.1 mm, and the test reproducibility was within 0.3 mm.

### 3.4 Comparison with a conventional isocenter verification test

The isocenter verification test and the conventional Winston–Lutz test was compared using the same linear accelerator. Displacements in the *X*, *Y*, and *Z* directions were measured, and the length of the displacement vector directed from the treatment isocenter to the imaging isocenter was calculated. The results agreed to within 0.4 mm (Table 6).

**TABLE 6:** Comparison between the 3D isocenter verification test and the conventional Winston–Lutz test.

	3D isocenter verification test (prescription dose of 20 Gy)	Winston‐Lutz test with alignment using kV‐CBCT
*X* (mm)	0.23	0.3
*Y* (mm)	0.09	–0.1
*Z* (mm)	–0.09	0.3
Vector (mm)	0.27	0.2

Abbreviations: kV‐CBCT, kilovoltage cone‐beam computed tomography; 3D, three dimensional.

## 4 DISCUSSION

In this study, we verified the robustness of the 3D treatment and imaging isocenter test using dGEL. Pant et al. found that the verification test did not depend on the CNR when detecting the beam axis in CT images.^7^ Our results using the dGEL dosimeter is consistent with those of Pant et al. using the NIPAM‐based dosimeter in that the displacements were within 0.5 mm for doses of 5–20 Gy. However, the length of the displacement vector at 5 Gy deviated from that at other doses by as much as 0.7 mm for field No. 6, where the beam axis was detected with a CNR of less than 1.0. We suggest that the CNR does not need to be very high; however, a low CNR could decrease the detection accuracy. The CNR should be affected by the image acquisition parameters (especially the tube potential, tube current, slice thickness, and number of projections) and image reconstruction parameters. CBCT imaging with long acquisition times and low gantry speeds permits a large number of projections to be acquired, resulting in a high CNR. Although we used one set of parameters in this study, optimizing them might improve the CNR or reduce the overall QA time. This study shows that the rapid response of the dGEL dosimeter is useful for achieving sufficient accuracy with a radiation dose of approximately 10 Gy. In terms of the detectability, the residual error of the test with the intentional error was within 0.2 mm. For the reproducibility, the analysis and image quality variations for a single dGEL resulted in discrepancies within 0.3 mm. Intra‐ and inter‐batch reproducibility of the dGEL are reported to be good in the 5–35 Gy range.^12^ This methodology should be robust when working with small measurement uncertainties.

In this study, the results of the Winston‐Lutz test and the 3D isocenter verification test agreed within 0.4 mm. The Winston–Lutz test was performed using an x‐ray beam energy of 2.5 MV, a clinically representative energy used for QA in our hospital. Using the Winston–Lutz test at various energies associated with different steering parameters of a tuned linear accelerator, Zhang et al. reported a 0.35 mm deviation among the measured displacement vector lengths.^13^ Considering such the uncertainty of energy difference, the agreement would be good. The current verification test may be interpreted in a manner similar to that of other tests, including the conventional Winston‐Lutz test. However, the conventional tests cannot visualize the beam axis in 3D, and the displacement obtained using the conventional verifications also includes the residual error of alignment and positioning of a detector, such as an electronic portal imaging device.

The test provides detailed information about a radiation beam. Even with the current sensitivity, the test may be used not only for monthly or annual QA but also during commissioning of new instruments or when a detailed investigation is required. The dGEL can perform the test without the need for other QA tools. It can also be stored at room temperature after arrival at the facility and is easy to handle.

In recent years, single‐isocenter irradiation of multiple brain metastases has been clinically implemented in SRS/SRT.^14^ The accuracy of off‐isocenter irradiation has been investigated, and several methods to verify this have been reported.^15^, ^16^, ^17^ In this study, the irradiation fields were set to intersect at the isocenter, but this test can theoretically be used to verify the accuracy of multiple off‐axis targets.

The methods used in our study, including irradiation, imaging, and analysis, resulted in a small (0.2–0.3 mm) measurement uncertainty. To create the image for the analysis, subtraction of the pre‐irradiation CBCT image from the post‐irradiation CBCT image was used to remove cupping artifacts. Thus, the positions of the pre‐ and post‐irradiation CBCT images must be consistent. In this study, non‐coplanar irradiation was used, which means that treatment couch movement may shift the dGEL itself. Such movement will not affect the accuracy of the measurement, but it will affect the image subtraction. In this study, the dGEL was immobilized using adhesive tape (Figure 4); thus, care needed to be taken to ensure the quality of the subtracted image.

A dose of 10 Gy or more is needed to visualize the beam trajectories on CBCT images using the new dGEL, which is much lower than that required for the NIPAM‐based dosimeter. Because a lower radiation dose provides better efficiency, the sensitivity of the dGEL is expected to be higher than other dosimeters.

Oxygen contamination of a polymer dosimeter can have a markedly detrimental effect on its performance. Being a polymer gel, a prolonged period between fabrication and irradiation increases the probability of such contamination. Thus, the dGEL should ideally be used immediately after receipt. Further development of the polymer dGEL and selection of a suitable container that is impervious to oxygen contamination are required to extend the expiration date of such dosimeters.

## 5 CONCLUSIONS

We directly evaluated the coincidence of the treatment and kV‐CBCT isocenter coordinate systems using the dGEL dosimeter. We showed that it can achieve sufficient accuracy (< 0.4 mm) with a radiation dose of approximately 10 Gy. Combined with CBCT image acquisition and analysis, the dGEL dosimeter enables misalignment of the radiation beam due to gantry and couch rotation to be visualized and quantified with a small measurement uncertainty of 0.2–0.3 mm.

## AUTHOR CONTRIBUTIONS

Riki Oshika implemented the in‐house program and collected and analyzed data. Hidenobu Tachibana conceptualized the study, developed the study design, supervised the authors throughout the study, and provided expertise in manuscript preparation. Seki Kazuya collected measurement data. Rie Tachibana fabricated the dGELs. Shunsuke Moriya and Takeji Sakae helped to analyze and interpret the data and critically review the manuscript.

## CONFLICT OF INTEREST STATEMENT

The authors declare no conflicts of interest.
